# Impact of Emotional Support, Informational Support, and Norms of Reciprocity on Trust Toward the Medical Aesthetic Community: The Moderating Effect of Core Self-Evaluations

**DOI:** 10.2196/11750

**Published:** 2019-03-21

**Authors:** Jyh-Jeng Wu, Haider A Khan, Shu-Hua Chien, Yu-Peng Lee

**Affiliations:** 1 Department of Business Management National United University Miaoli Taiwan; 2 Josef Korbel School of International Studies University of Denver Denver, CO United States; 3 Department of Insurance and Finance National Taichung University of Science and Technology Taichung Taichung Taiwan

**Keywords:** informational support, emotional support, norms of reciprocity, trust transfer theory, core self-evaluation

## Abstract

**Background:**

The consumption of medical aesthetic services has become popular in recent years. Many people have purchased medical aesthetic services and treatments in pursuit of self-beauty. When members of online medical aesthetic communities actively participate in discussions and encourage and support one another, there is an increase in community commitment, trust toward each other, and trust toward the community, ultimately promoting social sharing in an environment of positive feedback.

**Objective:**

This study aimed to explore via the theory of social support—grounded in a deeper social capabilities framework developed by Khan following the Nobel laureate Amartya Sen’s groundbreaking work—whether emotional support, informational support, and norms of reciprocity in online communities impact group members in terms of creating trust toward other members. This enhances trust toward the community and generates a sense of community commitment, ultimately impacting social buying intention and social sharing intention.

**Methods:**

This study used IBM SPSS and AMOS to analyze data. Data were collected through online questionnaires in online medical aesthetic community forums, thereby producing samples that were both representative and accurate. To understand whether core self-evaluation (CSE) is a moderator in the relationship between social sharing intention and social buying intention, this study averaged the point of CSEs in the sample after statistical analysis, dividing the sample into 2 groups.

**Results:**

The results showed that emotional support and norms of reciprocity positively impact trust toward members, and trust toward members positively impact trust toward the community. This generates trust transfer, which positively impacts social buying intention and social sharing intention. At the same time, CSE is a moderator variable between trust toward the community and social buying intention, but CSE is not a moderator variable between trust toward the community and social sharing intention.

**Conclusions:**

This study revealed that when members of online medical aesthetic communities actively participate in discussions and encourage and support one another, community commitment, trust toward each other, and trust toward the community increases, ultimately promoting social sharing and buying intentions.

## Introduction

### Background

The consumption of medical aesthetic services has become popular in recent years. Many people have purchased medical aesthetic services and treatments in pursuit of self-beauty. In 2017, the market size of global medical aesthetics was nearly US $230 billion. The growth of Taiwan’s medical aesthetic device market was ranked among the top 3 in Asia. Nearly 60% of Taiwan’s working class spent US $2300 to US $6700 on plastic surgery and microsurgery each year. On average, hyaluronic acid injections were used 193 times daily, and 30% of visitors from Mainland China came exclusively for *beauty* purposes. As such, the medical aesthetics industry contributes around US $1.4 billion to Taiwan’s gross domestic product and continues to rise.

As technology advances and online communities continue to prosper, consumers often search for answers and support from various online communities before making a decision to purchase [1]. When searching for answers and support from online communities, consumers’ trust toward other members and the community is a key factor impacting the decision to purchase [2].

According to the survey results of the *Word-of-Mouth Demand of Online Communities* conducted by the Market Intelligence and Consulting Institute, in 2014, nearly 81% of consumers searched the internet for word-of-mouth information via social network sites (45.8%), discussion forums (44.7%), and blogs (33.1%). At present, the exchange of word-of-mouth information among consumers in Taiwan remains concentrated on frequently visited social network sites. On the basis of product type, consumers choose channels with different levels of trust regarding word-of-mouth information. Therefore, businesses should understand consumers’ word-of-mouth demands and internet search habits and should treat such data as important reference when introducing new products or planning annual marketing strategies. Medical research institutes can effectively communicate and connect with more people via online communities [3]. The literature also indicates that consumers tend to believe in and are eager to obtain medical information from online communities [4].

On the basis of the trend described above, this study explored whether a sense of trust is generated in consumers of the medical aesthetics industry from the community support they experience before purchasing or sharing and whether this will ultimately increase their intention to share or purchase.

### Theories of Social Support and Social Capabilities

Social support theory explains how resources and help are acquired through interpersonal interaction within a community unit. There are 2 types of social support, namely structural and functional. Structural social support emphasizes community size, operating systems, relationship intensity among community members, and whether a sense of belonging to the community is generated after interpersonal interaction [5]. On the basis of relationship intensity, structural social support can be further classified as formal structural social support from organizations such as hospitals and schools and informal structural social support from intimate relationships such as families, friends, and peers [6].

Functional social support refers to the subjective feeling formed by an individual after interacting with others. The acquired support intensity is evaluated using the relationship, process, and results of interaction [7]. Support can be categorized as tangible support, informational support, emotional support, esteem support, and network support [8]. Furthermore, functional social support can be delineated as esteem support, instrumental support, informational support, and emotional support [9]. As subjective feelings vary between people, the definition of social support has not yet been standardized in academic research. However, consensus has been reached on the 3 major types of social support, namely tangible support, informational support, and emotional support [10] as the bases for further extension and adjustment.

Social support can also be regarded as a reward acquired from interpersonal interaction. In other words, social support can be viewed as resources acquired by an individual who has exchanged something with others based on the purpose or needs of the individual [11]; examples are tangible support such as monetary assistance and gifts [12] and intangible support such as caring, listening, and giving advice [1]. The higher the social support intensity an individual feels after interacting with others in a group, the higher the individual’s intention to establish an intimate interactive relationship in the group [13].

The theory of social support can be expressed in a more rigorous form by nesting it into a theory of social capabilities–based flexicurity in a learning economy. The basic idea has been developed by various theorists [14-24] who drew upon the insights of Adam Smith; they have proposed a theoretically rigorous and elaborate evaluation of well-being [25,26]. Sen is the originator of this *capability approach* in recent years [27]. The theoretical criticisms of the utilitarian approach by Sen et al—this approach reduces all qualities into the quanta of utilities—are serious ones. Khan et al has pursued a similar line of criticism in a number of recent papers and in his book *Technology, Development, and Democracy* [28-31]. This approach makes the capabilities explicitly social and asks what concatenation of institutions, both economic (real and financial) and other (eg, political and social), will allow social support and trust to increase steadily and at the same time equalize them among diverse individuals [32]. In effect, as the following discussion makes clear, we are asking how we can increase and equalize real positive freedom for individuals in specific social contexts—in this study, specifically for online communities and individuals. In his book, *Technology, Development and Democracy*, Khan points out that trust and freedom are interactive arrangements in a society where concrete institutions at many levels and technologies of production and exchange, all play definite roles [33]. The social support theory can be elaborated in a deeper way by building on these foundations and applying them dynamically to (post)modern networked online communities, with social capability–building through causal connections and positive feedback loop mechanisms. In this approach, methodologically, confirmatory factor analysis can support or disqualify the particular causal mechanisms that are proposed in this study and others in this area of research. We now turn to the specificities of social support in an online environment.

### Social Support

As the use of the internet has grown rapidly in recent decades, more people engage in online communications every day. In particular, as social network sites proliferate, easy exchanges among users are facilitated by the online environment, in which social support has gradually transformed into social support communication [14]. Online social support refers to information and emotional exchanges in the virtual space and is provided through informational support and emotional support [15]. Social support and online communities are closely related [16]. Previous studies showed the connectedness between social support and trust [17]. In this study, we believed that informational support and emotional support benefit the relationships of all related parties and are conducive to relationship building and maintaining a good online environment.

### Emotional Support

Emotional support refers to demonstrating to others empathy, care, love, understanding, or encouragement [18], making them feel they are being given attention. In contrast to informational support, emotional support emphasizes the emotional side of social support, which may indirectly help overcome problems [34]. Emotional support enables community members to obtain help from other members. Specifically, love is the foundation for developing trust [18]. Therefore, people develop community exchanges and trust through emotional exchange and contact with other members in the community. In addition, if a member obtains love and warmth from other members in the group who have similar experiences of pain and challenges, he/she will often feel more comfortable remaining in the focus group. This is because emotional responses such as love and care satisfy members’ needs for respect and social attention, enabling them to identify as members of the group with a sense of belonging [35].

### Informational Support

Informational support provides individuals with advice, guidance, or useful information that helps them solve problems, generate new ideas, or make good decisions [36]. If people continue receiving positive help such as valuable recommendations or instant help from network friends or online groups, they are more likely to show benevolence, integrity, and capability to the other party, further enhancing their trust toward members who provided the related information [37].

As mentioned, interest in a relationship is necessary in the development of cooperative relationships [38]. Therefore, if group members can benefit from others’ opinions, they are likely to agree on the values of the focus group and maintain a long-term relationship with it.

### Norms of Reciprocity

Norms of reciprocity are behavior norms in a social community that obligate people who have received help from others in the community to offer similar help in return in the future [39,40]. When community norms are gradually transformed into individual values in a community, members consider exchange behavior based on a friendly interactive relationship as natural, which impacts their sharing intention in the future [41]. Thus, the higher the intensity of norms of reciprocity, the higher will the frequency of exchange activities be [42]. Furthermore, members’ knowledge collection behavior is enhanced if the knowledge provided on knowledge sharing platforms is reliable and comprehensive [2]. As such, the more the knowledge is aligned to members’ needs, the more willing they will be to offer useful knowledge to other members to help them resolve their problems. Previous studies indicated that norms of reciprocity promote trust in a community [43]. Thus, we hypothesized that norms of reciprocity have a significant positive impact on trust toward members.

### Trust

Trust is considered a basic factor in the formation of successful relationships [38]. In particular, recent studies focused on the relationship between trust toward providers of products and services and customers’ online purchase intention [44]. As retrieving information from the internet becomes more popular, consumers ask for recommendations from social communities and people they trust. On the contrary, privacy issues mean that consumers are more likely to share their own information with someone they trust [45]. Past studies also confirmed the relationship between brand community and consumer participation [46]. Therefore, the impact of trust on social sharing intention and social buying intention—as well as trust toward members and the community—is discussed in this study.

### Trust Toward Members

In this study, trust toward members is defined as an individual’s intention to rely on and believe in other community members, such as believing the information they provide and taking action and making decisions based on other members’ recommendations. Previous studies found that trust toward members impacts online participation behavior such as seeking and providing information in focus groups [47], because in an environment with mutual trust, people often help each other, which develops into common community activities. In particular, information obtained from credible sources is often considered more useful and treated as the basis for decision making [48]. In addition, people are more likely to share their consumption experience of products or services afterward.

When other members display reliable characteristics of the individuals seeking information, such as benevolence, integrity, capableness, or have had a similar experience, information-seeking members feel comfortable talking to one another as they share a common knowledge background. As such, any suspicion or doubts in each other are alleviated.

**Figure 1:**
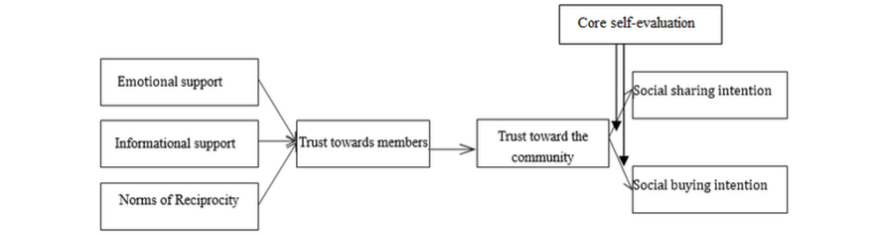
Research model.

### Trust Toward the Community

Trust toward the community refers to the extent to which people rely on and trust a community. Generally accepted standards are enforced by online communities to instill norms of reciprocity among their members. In addition, benevolence and integrity in a community can lower doubts among members. The relationship between trust toward the community and customer loyalty has been confirmed [49]. Essentially, the stronger the trust an individual has for a community, the higher the tendency that individuals will seek advice from that community on products or services they are interested in buying and share their consumption experience within the community afterward.

### Community Commitment

Relationship commitment reflects many aspects of the long-term relationship [38]. Previous studies confirm the relationship between commitment and users [36], indicating that when users are committed to an online community, they contribute and participate more in the online community [50]. Previous studies also found that online brand community commitment significantly impacts consumers’ decision making [51]. In an online business community environment, the network platform provides a shared space within which users can exchange information and communicate. Users can share their consumption experiences with friends in online communities or seek product recommendations from their network of friends. If users are committed to maintaining a sustainable commitment relationship with an online community, they will strive to maintain this commitment and are more likely to participate in various group activities to help grow the community.

### Trust Transfer Theory

According to the trust transfer theory, interpersonal trust develops into trust toward the community for 2 reasons. First, trust among members makes users believe that information provided in the community is more credible, which reassures members that the community will continue to improve its service quality and provide effective management in building a credible communication environment [52]. Second, previous studies pointed out that trust among members increases members’ trust toward the community [53]. Previous studies reported that when people trust sellers in a Business to Consumer online community, their trust could be transferred to the related applications [54]; to give an example, Asia’s famous and successful retail drugstore brand, Watsons, has its own online community and app. Other studies confirmed the relationship of trust transfer between brand community and consumer participation [46]. When members trust one another, a bigger and stronger community is created, enabling members to consider the community a suitable venue for communication.

### Core Self-Evaluation

In the past, much research focused on core self-evaluation (CSE) when studying different kinds of human behavior, for example, the relationship between CSE and social support [55]. CSE is defined as the basic evaluation of one’s own values, potential, capabilities, and talents [56]. This study hopes to understand the mediating role of CSE on social sharing and buying intentions. Therefore, we hypothesized that CSE is a moderator variable between trust toward the community and social sharing and buying intentions.

In sum, this study explored whether emotional support, informational support, and norms of reciprocity have an impact on social sharing and social buying intentions through trust toward members and trust toward the community using CSE as the moderator variable. The framework of the study is provided in Figure 1.

## Methods

The participants in the pretest were members in online medical aesthetics communities. A total of 50 questionnaires were retrieved and after selection, a consistent scale was developed for informational support, emotional support, norms of reciprocity, trust toward members, trust toward the community, and CSE. The questionnaire was created using Google Forms. The link to the questionnaire was disseminated on Facebook and LINE, which are among the most popular forms of social media in Taiwan. The questionnaires were placed in online medical aesthetics communities with the hope of collecting data approaching that of the population and understanding the true focus and recognition of online medical aesthetics communities’ users, thereby producing samples that were both representative and accurate. Using previously validated instruments, questionnaire items based on a 7-point Likert scale ranging from 1 (*Strongly Disagree*) to 7 (*Strongly Agree*) were borrowed from the literature and adapted to this study to measure the constructs. To address measurement concerns, this study referenced the research of past scholars regarding each dimension.

## Results

### Demographics

The questionnaires were released on January 11 to 31, 2018, and a total of 565 valid questionnaires were collected. Before we started the survey, we first contacted 800 online medical aesthetics community users and asked if they were willing to participate in the survey. A total of 780 online users indicated a willingness to take part in the survey. We then sent questionnaires to those users. The returned surveys yielded a response rate of 78.2% (610/780). After deleting 45 incomplete responses (eg, respondents answered *Strongly Disagree* or *Strongly Agree* to all questions), a total of 565 valid observations were collected (a response rate of 70.6%). Table 1 shows the demographic characteristics of the participants. Most of the samples were female, aged 18 years older, and most of them were university graduates. Most of the samples had less than 2 years of community experience, and most of them were willing to spend less than US $10,000 on medical aesthetics.

### Reliability and Validity Tests

We analyzed data using SPSS 12 and AMOS 22. The measurement model fit indices (v2/*df*=2.39; root mean square error of approximation, RMSEA=0.05; confirmatory fit index, CFI=0.982; adjusted goodness of fit index, AGFI=0.906; and Bollen fit index, IFI=0.982). The most widely used incremental fit index, CFI, has a value of 0.982, which exceeds the guideline value of 0.90 for a model of this complexity and size. This model has not been compared with other models; however, the value of parsimony index AGFI (0.90) reflects a good model fit. All these absolute, incremental, and parsimony fit indices suggest an acceptable fit for the measurement model.

Table 2 shows the results of reliability and validity testing. Regarding the reliability of the question items, the factor loading of all the items ranged between 0.7 and 0.95. With their factor loading values being greater than 0.5, these items exhibited high reliability. Average variance extracted (AVE) testing was used to assess the convergent validity of the items. It demonstrated that the set of indicators had an AVE greater than the 0.5 threshold. The composite reliability (CR) of measured variables was estimated to test their internal consistency in the measurement model. CR indicates how well these variables represent latent variables; a CR value of greater than 0.7 generally shows high internal consistency. In this study, the CR values of all constructs in the corrected model ranged between 0.89 and 0.96, indicating that the question items of the constructs had sufficient reliability.

**Table 1:** Respondent profiles.

Measure		Frequency	Statistics, n (%)
**Gender**			
	Male	134	23.7
	Female	431	76.3
**Age**			
	<18	5	9
	19-30	308	54.5
	>31	252	44.6
**Education**			
	Junior high school	13	2.3
	High school	124	21.9
	College or postgraduate	428	75.8
**Usage experience of online community**			
	<1 year	244	43.2
	1-2 years	75	13.3
	2-3 years	72	12.7
	3-4 years	43	7.6
	>5 years	131	23.2
**Willingness to spend on medical aesthetics (US $)**			
	<10,000	353	62.5
	10,000-50,000	110	19.5
	50,000-100,000	66	11.7
	>100,000	36	6.4

**Table 2:** Results of confirmatory factor analysis.

Constructs		Loadings	Square Multiple Correlation
**CSEs^a^ (CR=0.962; AVE=0.710; Cronbach alpha=.960; Judge et al [56])**			
	I am confident I get the success I deserve in life.	.772	.596
	When I try, I generally succeed.	.797	.635
	Usually, I feel happy.	.829	.687
	When I fail, I don’t feel worthless.	.813	.661
	I complete tasks successfully.	.886	.785
	Usually, I feel in control of my work.	.876	.767
	Overall, I am satisfied with myself.	.884	.781
	I am filled with confidence about my competence.	.879	.773
	I determine what will happen in my life.	.785	.616
	I feel in control of my success in my career.	.864	.746
	I am capable of coping with most of my problems.	.863	.745
	There are times when things look pretty hope to me.	.849	.721
**Emotional support (CR=0.922; AVE=0.866; Cronbach alpha=.903; Liang et al [36])**			
	When faced with difficulties, some people on this website are on my side with me.	.910	.828
	When faced with difficulties, some people on this website comforted and encouraged me.	.944	.891
	When faced with difficulties, some people on this website expressed interest and concern in my well-being.	.937	.878
**Informational support (CR=0.940; AVE=0.893; Cronbach alpha=.952; Liang et al [36])**			
	On this website, some people would offer suggestions when I needed help.	.939	.882
	When I encountered a problem, some people on this website would give me information to help me overcome the problem.	.950	.903
	When faced with difficulties, some people on this website would help me discover the cause and provide me with suggestions.	.946	.895
**Norms of reciprocity (CR=0.896; AVE=0.828; Cronbach alpha=.953; Chen and Hung [2])**			
	I believe that members of this shared platform will help each other.	.912	.832
	I should maintain a good interaction with the members of this shared platform.	.914	.835
	In this shared platform to trade goods or services, both parties can meet the demand.	.903	.815
**Trust toward members (CR=0.934; AVE=0.884; Cronbach alpha=.961; Liang et al [36])**			
	Members on this website will always try and help me out if I get into difficulties.	.931	.867
	Members on this website will always keep the promises they make to one another.	.951	.904
	Members on this website are truthful in dealing with one another.	.939	.882
**Trust toward the community (CR=0.941; AVE=0.894; Cronbach alpha =.973; Liang et al [36])**			
	The performance of this website always meets my expectations	.944	.891
	This website can be counted on as a good social networking site.	.946	.895
	This website is a reliable social networking site.	.947	.897
**Social sharing intention (CR=0.935; AVE=0.886; Cronbach alpha=.966)**			
	I am willing to provide my experiences and suggestions when other members on this website want my advice on micro cosmetic surgery.	.941	.885
	I am willing to share my own micro cosmetic surgery experience with other members on this website.	.953	.908
	I am willing to recommend micro cosmetic surgery that is worth buying to other members on this website.	.929	.863
**Social buying intention (CR=0.892; AVE=0.823; Cronbach alpha=.874; Liang et al [36])**			
	I will consider the experiences of other members on this website when I want to have micro cosmetic surgery.	.914	.835
	I will ask other members on this website to provide me with their suggestions before I have micro cosmetic surgery.	.932	.869
	I am willing to have micro cosmetic surgery that recommended by other members on this website.	.875	.766

^a^CSE: core self-evaluation.

**Table 3:** Pearson correlation coefficients.

Pearson correlation coefficient constructs	1	2	3	4	5	6	7	8
1. Emotional support	(.930)	—^a^	—	—	—	—	—	—
2. Informational support	.791^b^	(.944)^c^	—	—	—	—	—	—
3. Norms of reciprocity	.750^b^	.782^b^	(.909)	—	—	—	—	—
4. Trust toward members	.742^b^	.673^b^	.764^b^	(.940)	—	—	—	—
5. Trust toward the community	.679^b^	.662^b^	.746^b^	.849^b^	(.945)	—	—	—
6. Social sharing intention	.615^b^	.675^b^	.644^b^	.629^b^	.645^b^	(.941)	—	—
7. Social buying intention	.605^b^	.666^b^	.675^b^	.595^b^	.666^b^	.717^b^	(.907)	—
8. Core self-evaluations	.400^b^	.417^b^	.445^b^	.391^b^	.387^b^	.471^b^	.397^b^	(.842)

^a^Not applicable.

^b^*P*<.01.

^c^Parentheses signify the square root of each construct’s average variance extracted.

**Table 4:** Path coefficients.

Hypothesis testing	Path value	CR	*P* value	Research assessment result
Emotional support → Trust toward members^a^	0.347	6.094	—^b^	Yes
Informational support → Trust toward members	–0.151	–2.222	.026	No
Norms of reciprocity → Trust toward members^a^	0.695	10.146	—	Yes
Trust toward the community → Social sharing intention^a^	0.932	19.144	—	Yes
Trust toward the community → Social buying intention^a^	0.849	18.466	—	Yes
Trust toward members → Trust toward the community^a^	0.890	28.528	—	Yes

^a^*P*<.001.

^b^Not applicable.

Table 3 tabulates the Pearson’s correlation coefficients of the variables. Significant positive correlations were observed among emotional support, informational support, norms of reciprocity, trust toward members, trust toward the community, social sharing intention, social buying intention, and CSEs. In addition, all constructs achieved discriminant validity, because the square root of each construct’s AVE was higher than its correlation between any of the constructs [57].

This study performed a structural equation model analysis using Amos 22. The model fit indices (v2/*df*=2.39; RMSEA = 0.05; CFI = 0.98; AGFI = 0.906; and IFI = 0.982) suggested that the model represents a satisfactory fit to the data [58]. The model fit indices supported a good model fit. Therefore, further analyses of the relationships among the modeled constructs were conducted.

**Figure 2:**
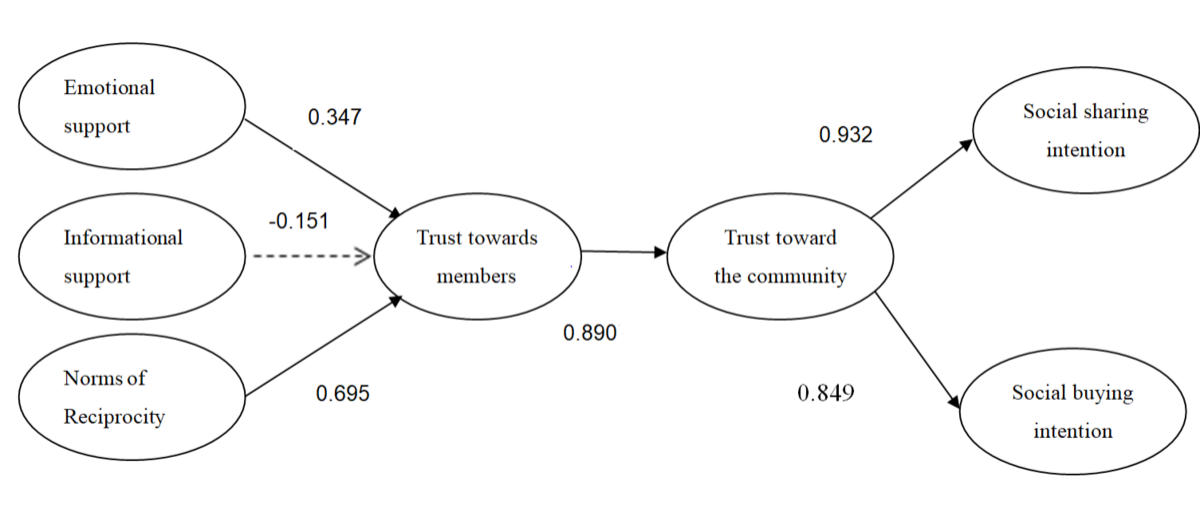
Results of research model. Numbers represent path coefficients.

The results showed that emotional support and norms of reciprocity positively impact trust toward members, and trust toward members positively impact trust toward the community. This generates trust transfer, which positively impacts social buying and sharing intentions. However, the informational support did not have a significant effect on trust toward members, possibly demonstrating consumer distrust toward medical advertising. The path coefficients of hypothesis testing are summarized in Figure 2 and Table 4.

After all the hypotheses were validated, a Sobel test was conducted to assess mediating effects among variables. A value lower than 0.05 in AMOS and a z score of greater than 1.96 in the Sobel test results indicate significant mediating effects [59]. The Sobel test results showed that emotional support affected trust toward the community through trust toward members (Sobel z=5.99); norms of reciprocity affected trust toward the community through trust toward members (Sobel z=9.55); trust toward members affected social sharing intention through trust toward the community (Sobel z=16.03); trust toward members affected social buying intention on the basis of trust toward the community (Sobel z=15.45). In sum, trust toward members mediated the effects of emotional support and norms of reciprocity on trust toward the community, whereas trust toward the community mediated the effects of trust toward members on social sharing intention and social buying intention.

To understand whether CSE is a moderator on the impact of social sharing and buying intentions, this study averaged the point of CSEs in the sample after statistical analysis and, referring to the past research methods [60], this study divided the sample into 2 groups: high CSE (Average point of CSE>4.85) and low CSE (Average point of CSE<4.85), and compared the group differences based on the past research [61]. Table 5 shows that CSE is a moderator variable between trust toward the community and social buying intention, but CSE is not a moderator variable between trust toward the community and social sharing intention. The path coefficients of hypothesis testing are summarized in Table 6.

**Table 5:** Path coefficients of hypothesis testing by group differences.

Hypothesis testing by group differences	High core self-evaluation		Low core self-evaluation		Z score
	Estimate	*P* value	Estimate	*P* Value	
Emotional support → Trust toward members	0.366	.000	0.301	.000	–0.604
Informational support →Trust toward members	–0.174	.058	–0.163	.105	0.081
Norms of reciprocity → Trust toward members	0.784	.000	0.703	.000	–0.541
Trust toward members → Trust toward the community	0.948	.000	0.918	.000	–0.411
Trust toward the community → Social sharing intention	0.744	.000	0.868	.000	1.214
Trust toward the community → Social buying intention	0.682	.000	1.111	.000	3.748^a^

^a^*P* value<.01.

**Table 6:** Path coefficients.

Compare groups		Path Value	CR	*P* value	Research assessment result
**High CSE^a^**					
	Trust toward the community → Social sharing intention	0.858	14.724	<.01	Y
	Trust toward the community → Social buying intention	0.806	12.114	<.01	Y
**Low CSE**					
	Trust toward the community → Social sharing intention	0.742	9.757	<.01	Y
	Trust toward the community → Social buying intention	0.915	11.139	<.01	Y

^a^CSE: core self-evaluation.

## Discussion

The topic of CSE is widely discussed in the field of human resources, but not many scholars have applied it to the discipline of the medical industry. This study introduces CSE into a research framework to investigate the social sharing and buying intentions of members of medical aesthetics communities. Furthermore, this study contributed to a theoretical deepening of the approach by grounding it into a causally deeper social capabilities framework developed by Sen et al.

Thus, grounded theoretically, the results of the study showed that when members of medical aesthetics communities experience emotional support, their trust toward other members strengthen and trust transfer occurs, which reinforces trust toward the community and enhances commitment. Similarly, when norms of reciprocity among community members are high, members’ trust toward other members also strengthen, bringing about trust transfer, which reinforces their trust toward the community and increases commitment.

In addition, CSE is a moderator on the impact of social buying intention. The relationship between social support and trust can be explained to a large extent by considering the deeper causal relations between community norms and individual values [62]. When community norms gradually transform into individual values, community members come to consider the exchange behavior built on friendly interactive relationships as natural, which impacts future sharing intention behavior. This study also revealed empirically that when members of online medical aesthetics communities actively participate in discussions and encourage and support one another, community commitment, trust toward each other, and trust toward the community increases, ultimately promoting social sharing intentions in a positive feedback loop.

This study explores consumers’ social sharing and buying intentions for medical aesthetic products or services and the relationship among variables. Therefore, the medical aesthetics industry can use this study to understand the thoughts and needs of the public to facilitate more effective marketing strategies for related online communities.

For doctors, after surgery, they can encourage patients to share what they liked about the surgery in the online community, who will in turn earn discounts for their next purchase. By doing so, the happy patients will feel confident and satisfied based on the attention of other members in the online community. For patient education, the medical aesthetics industry can increase trust from its customers through recommendations from other network friends who have had positive experiences of the surgery, which can lead to a positive feedback loop of growing trust. For population health or clinical care, the medical aesthetics industry has grown so much that competition is intensifying. Our results are consistent with the normative prescription that practitioners in the medical aesthetics industry offer discount programs as incentives for customer sharing in online communities. From our result, the medical aesthetics industry can support a public forum to provide trust, emotional support, and informational support to forum members, because trust toward the community positively affects social buying and sharing intentions.

In the end, we focused on the medical aesthetics industry, which has grown rapidly in recent years. Future studies can explore whether the same causal and empirical analysis applies to online communities in other growing industries. As the number of online community platforms has grown in recent years, future studies could explore different platforms and compare the results to provide a wider spectrum of contributions to the field of online communities.

## Abbreviations

AGFI: adjusted goodness of fit index
AVE: average variance extracted
CFI: confirmatory fit index
CR: composite reliability
CSE: core self-evaluation
IFI: Bollen fit index
RMSEA: root mean square error of approximation
